# Cyclical ectopic Cushing’s syndrome due to a mediastinal neuroendocrine tumor: a case-based review

**DOI:** 10.1007/s11845-026-04432-w

**Published:** 2026-05-13

**Authors:** Carolina Peixe, Marta Vaz Lopes, Mariana de Griné Severino, Ana Coelho Gomes, Ema Lacerda Nobre

**Affiliations:** 1 Endocrinology Service, Hospital de Santa Maria, Unidade Local de Saúde de Santa Maria, Lisbon, Portugal; 2 Faculty of Medicine, University of Lisbon, Lisbon, Portugal

**Keywords:** Cyclical Cushing’s syndrome, ACTH-dependent Cushing’s syndrome, Ectopic ACTH secretion, Neuroendocrine tumor, Hypercortisolism, Bilateral adrenalectomy

## Abstract

**Background:**

Cyclical Cushing’s syndrome (CCS) is an uncommon form of endogenous hypercortisolism characterized by alternating periods of cortisol excess and remission. Its intermittent nature delays diagnosis and localization of the adrenocorticotropic hormone (ACTH) source and complicates therapeutic decision-making.

**Case presentation:**

We report a 46-year-old man with an incidentally detected anterior mediastinal mass, diagnosed as a well-differentiated neuroendocrine tumor (NET) with ectopic ACTH production. He presented with clinical and biochemical features of ACTH-dependent Cushing’s syndrome. Despite tumor resection and multiple subsequent therapies, including somatostatin analogues, chemotherapy (without concomitant glucocorticoids), and radiotherapy, the disease course was marked by recurrent peaks of hypercortisolism interspersed with partial remissions, consistent with CCS. Episodes were associated with hypertension, diabetes mellitus, hypokalemia, infections, and thromboembolism. Due to refractory disease, bilateral adrenalectomy was performed, achieving biochemical remission but requiring lifelong glucocorticoid replacement.

**Discussion:**

CCS poses significant diagnostic and therapeutic challenges. Intermittent cortisol secretion may obscure diagnosis during remission phases and complicate assessment of treatment response. In NET-related CCS, tumor progression and therapeutic interventions may influence ACTH secretion, contributing to cyclical patterns. The “timing paradox”, whether to treat during peaks or remission, remains a key clinical dilemma.

**Conclusion:**

CCS requires prolonged biochemical surveillance, early recognition of cyclicity, and multidisciplinary management. The cumulative burden of recurrent hypercortisolism highlights the need for individualized strategies integrating endocrine and oncologic care. Bilateral adrenalectomy remains a life-saving option when sustained control is not achieved.

## Introduction

Cyclical Cushing’s syndrome (CCS) is a rare and diagnostically challenging variant of endogenous Cushing’s syndrome, characterized by recurrent episodes of cortisol excess interspersed with periods of biochemical remission or even hypocortisolism [1, 2]. This intermittent hormonal pattern complicates both diagnosis and management, often delaying definitive treatment and contributing to cumulative morbidity [1, 3]. Reported prevalence varies between 8% and 19% among patients with Cushing’s syndrome, depending on the diagnostic criteria applied, which are not yet standardized [1].

Both adrenocorticotropic hormone (ACTH)-dependent and ACTH-independent etiologies may follow a cyclical course. Pituitary corticotroph adenomas (Cushing’s disease) are the most frequent cause, followed by ectopic ACTH secretion — commonly from neuroendocrine tumors (NETs) — and, less frequently, adrenal causes, including micronodular adrenal hyperplasia (particularly primary pigmented nodular adrenocortical disease (PPNAD)) and autonomous adrenal adenomas [3, 4]. The pathophysiology of CCS is not yet fully understood but likely reflects complex interactions between tumor biology, hypothalamic–pituitary–adrenal (HPA) axis regulation, and treatment-related factors [2, 3, 5].

Recognition of CCS is crucial because untreated recurrent hypercortisolism carries significant risks, including metabolic syndrome, cardiovascular disease, osteoporosis, opportunistic infections, and thromboembolic events [1, 3]. Moreover, the cyclical nature of cortisol secretion can mask biochemical confirmation during troughs and interfere with the assessment of therapeutic efficacy, particularly in NET-associated cases [4].

In this article, we present a case of ectopic ACTH secretion due to a mediastinal NET with a well-documented cyclical cortisol pattern, refractory to both hypercortisolism-directed medical therapy and oncologic treatment, ultimately requiring bilateral adrenalectomy.

In addition to presenting this case, we provide a structured case-based review of the literature, focusing on key aspects of cyclical Cushing’s syndrome relevant to clinical practice.

## Case report

A 46-year-old male was referred to our Endocrinology department in 2018 after the incidental finding of a large anterior mediastinal mass (127 × 96 mm) on chest computed tomography, performed during evaluation for persistent cough. Transthoracic biopsy revealed a well-differentiated (G2) NET with ACTH immunopositivity.

Retrospectively, the patient reported progressive lower limb edema, fatigue, and proximal muscle weakness over the preceding months. These symptoms, together with new-onset hypertension and hypokalemia raised the suspicion of Cushing’s syndrome.

Biochemical testing confirmed ACTH-dependent hypercortisolism: serum cortisol 62.3 µg/dL (normal: 6.2–18.0), ACTH 246 pg/mL (normal: 7–63), 24-hour urinary free cortisol (UFC) 3510 µg/24 h (normal: 167–827), with loss of circadian rhythm and absence of suppression after dexamethasone testing. Pituitary function was otherwise preserved. Additional findings included elevated chromogranin A (1875 ng/mL) and primary hyperparathyroidism — calcium 11.3 mg/dL (normal: 8.6–10.2), parathyroid hormone (PTH) 96.9 pg/mL (normal < 72) — associated with osteopenia documented by DXA (Z-score: femur − 2.0; L1–L4 − 1.9).

Given the coexistence of NET and primary hyperparathyroidism, multiple endocrine neoplasia type 1 (MEN1) was suspected, but genetic testing was negative.

In July 2018, the patient underwent surgical resection of the mediastinal mass. Pathology confirmed a G2 NET (Ki-67: 10%) with ACTH-positive immunoreactivity, consistent with an ectopic source of ACTH production. Despite resection, locoregional disease persisted.

Postoperatively, treatment with long-acting octreotide induced biochemical remission and stable disease for approximately one year (UFC: 215 µg/24 h; ACTH: 40.3 pg/mL). In late 2019, disease progression required additional therapies, including CAPTEM (capecitabine plus temozolomide), carboplatin–etoposide, CAPOX (capecitabine plus oxaliplatin) and radiotherapy (30 Gy).

Between 2020 and 2022, the patient had three major episodes of hypercortisolism, each emerging shortly after changes in oncological therapy or evidence of tumor progression. These phases of hypercortisolism were characterized by severe hypertension, new-onset or worsening diabetes mellitus, hypokalemia, recurrent infections (including influenza A pneumonia), one episode of pulmonary embolism, and biochemical evidence of hepatocellular and cholestatic liver injury, without evidence of structural liver disease on imaging. At the most severe exacerbation, UFC reached 7365 µg/24 h and ACTH 154 pg/mL.

Medical management achieved only partial disease control and included antihypertensive and antidiabetic agents, as well as metyrapone, titrated up to 1500 mg/day. Ketoconazole was contraindicated due to active liver disease, and osilodrostat was not available.

Disease progression was predominantly locoregional, with progressive increase in tumor burden at the primary site, and no clear evidence of widespread metastatic disease. Notably, periods of tumor growth or modification of oncological therapy were temporally associated with exacerbations of hypercortisolism, suggesting a close interaction between tumor activity and ACTH secretion.

Given refractory disease, progressive comorbidities (osteopenia, recurrent infections, nephrolithiasis — in the context of primary hyperparathyroidism — thromboembolic events, metabolic syndrome), and limited oncological options — including disease progression despite multiple lines of systemic therapy and lack of further effective or tolerable treatment alternatives — bilateral adrenalectomy was considered the best option and was performed in 2023. Although surgical criteria for primary hyperparathyroidism were met, a conservative approach was adopted, with bisphosphonate therapy, in the context of the patient’s overall oncological condition and limited life expectancy.

Postoperatively, the patient achieved sustained biochemical remission, requiring lifelong glucocorticoid and mineralocorticoid replacement therapy. He remains under palliative care for NET, with symptom-directed support.

**Fig. 1:**
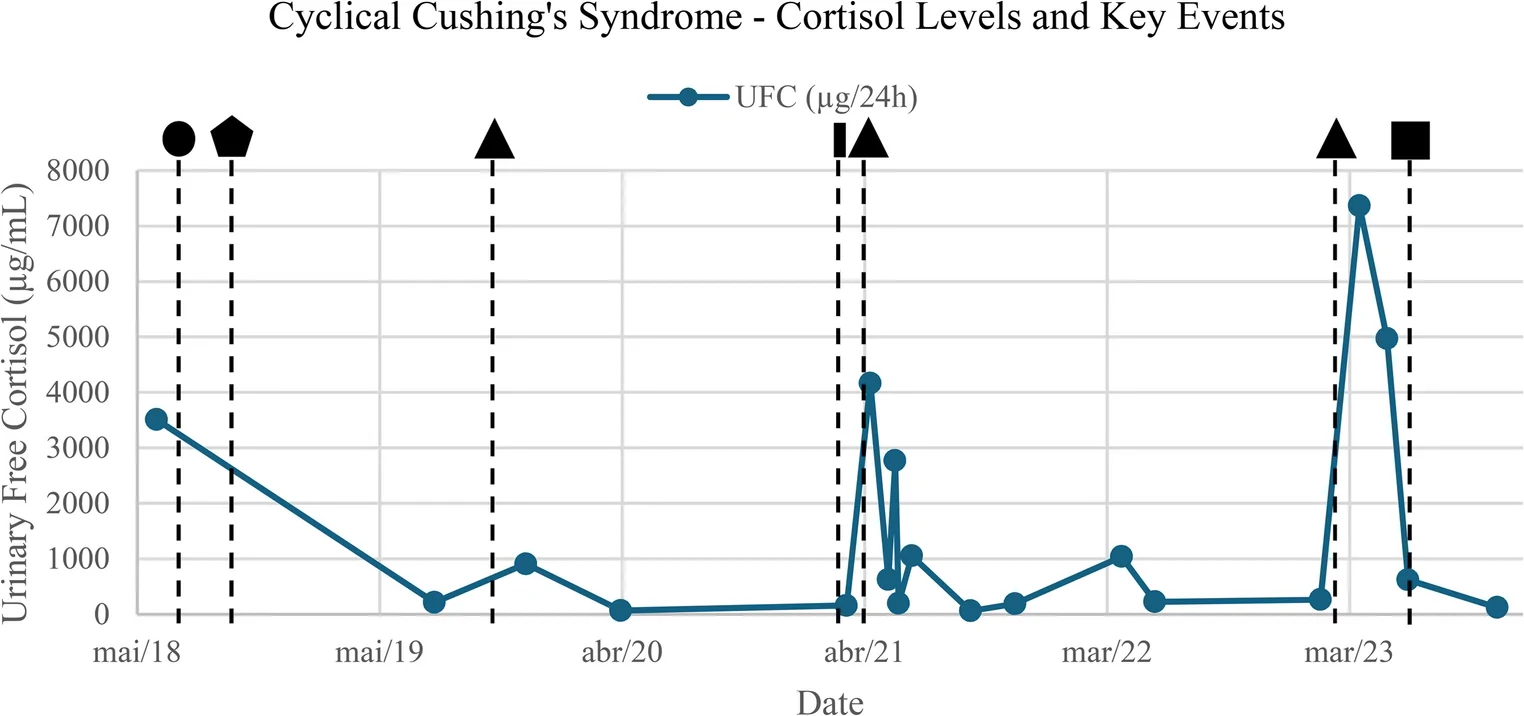
Evolution of UFC levels over time in relation to therapeutic interventions. Peaks in UFC reflect episodes of cyclical hypercortisolism, with partial biochemical control achieved transiently after surgical, medical, chemotherapeutic, and radiotherapeutic treatments. The persistent relapsing pattern ultimately led to bilateral adrenalectomy in late 2023. ● Mediastinal tumor resection (July 2018). ⬟ Octreotide (started October 2018). ▲ Chemotherapy (started December 2019 - CAPTEM, June 2021 - carboplatin–etoposide and March 2023 - CAPOX). ▮ Radiotherapy (started April 2021). ■ Bilateral adrenalectomy (September 2023)

The temporal relationship between UFC fluctuations and major therapeutic interventions is illustrated in Fig. 1, highlighting the cyclical nature of hypercortisolism and its association with tumor progression and treatment changes.

## Case-based review of the literature

### Overview of cyclical Cushing’s syndrome

#### Epidemiology and etiology

CCS is characterized by repeated episodes of excess cortisol separated by periods of normocortisolism or even hypocortisolism. Classical diagnostic definitions require three peaks and two troughs of cortisol secretion, although some authors accept two peaks and one trough, which increases the apparent prevalence. Depending on the criteria applied, between 8% and 19% of patients with endogenous Cushing’s syndrome demonstrate a cyclical pattern [5, 6].

In published series, the etiological distribution is broadly similar to that of Cushing’s syndrome overall, though with different proportions: approximately 54% of cases are due to Cushing’s disease, ~ 26% to ectopic ACTH secretion, and ~ 11% to adrenal causes, with the remainder unclassified [1, 2]. CCS has been reported across all age groups but is more frequently described in adults, with a slight female predominance in mixed-etiology cohorts. Ectopic ACTH secretion is particularly relevant in older patients and in those with rapidly progressive disease [3].

Both ACTH-dependent (pituitary and ectopic) and ACTH-independent (adrenal) forms may follow a cyclical course. In addition to pituitary adenomas and ectopic ACTH secretion, several adrenal conditions — including micronodular adrenal hyperplasia (notably PPNAD) and autonomous adrenal adenomas — have been associated with intermittent hypercortisolism. This highlights the importance of considering CCS across the full spectrum of etiologies, rather than limiting its recognition to pituitary disease [7].

#### Pathophysiological hypotheses

The mechanisms underlying cortisol cycling are heterogeneous and likely multifactorial:

- Tumor-intrinsic dynamics: Episodic ischemia/necrosis, fluctuations in secretory activity, and modulation of receptor or second messenger pathways within corticotroph or NET cells may produce intermittent ACTH release [1].
- HPA axis rhythmicity: Disruption of the normal coupling between circadian, ultradian, and infradian rhythms of the hypothalamic–pituitary–adrenal axis may contribute to pathological oscillations in cortisol secretion. In susceptible ACTH-secreting tumors, this misalignment can amplify normal physiological fluctuations, leading to cyclical hypercortisolism [1].
- Therapy-related effects: Antitumor treatments (e.g., somatostatin analogues, chemotherapy, radiotherapy) can transiently alter tumor viability or receptor expression (e.g., somatostatin receptor subtypes), leading to fluctuating cortisol secretion ACTH output — a phenomenon particularly reported in NET-related ectopic disease [3].
- Feedback and receptor biology: Dynamic changes in glucocorticoid feedback sensitivity, steroidogenic enzyme activity, and adrenal responsiveness may contribute to abrupt transitions between hyper- and normocortisolism [2].

Overall, CCS reflects complex interactions between tumor biology and HPA axis regulation, sometimes further modulated by therapeutic interventions.

#### Clinical implications

The cyclical nature of cortisol secretion in CCS poses significant challenges for both diagnosis and management. Because biochemical results may be normal during trough phases, serial testing — such as repeated 24-hour UFC or late-night salivary cortisol measurements — is preferred over single time-point dexamethasone suppression tests. Current Endocrine Society guidelines recommend repeated assessment when discordant results or strong clinical suspicion are present [7].

Dynamic testing, including suppression or stimulation protocols, may yield misleading results if performed during spontaneous nadirs or peaks, making prolonged biochemical surveillance essential to document the cyclic pattern [1].

In ectopic CCS, the source of ACTH secretion may be small, occult, or intermittently active, requiring a multidisciplinary diagnostic approach that combines biochemical confirmation, anatomical and functional imaging, and selective venous sampling when indicated. Initial localization often fails, and long-term follow-up is frequently necessary [3, 8].

Clinical severity can fluctuate dramatically. During cortisol peaks, patients face a heightened risk of opportunistic infections, thromboembolism, hypokalemia, hypertensive crises, and metabolic decompensation. In such instances of severe or “malignant” hypercortisolism, rapid stabilization with medical cortisol-lowering therapy, including metyrapone, ketoconazole, or etomidate, is essential. Bilateral adrenalectomy should be considered when medical control is inadequate — an approach that can be lifesaving in ectopic or NET-related disease [9, 10].

Finally, the so-called “timing paradox” — whether to intervene during peaks or await biochemical quiescence — complicates both endocrine and tumor-directed strategies. Close, prospective monitoring allows alignment of therapeutic windows with disease activity and facilitates interpretation of responses to NET-directed regimens, which may themselves influence cortisol cycling [2].

### Diagnostic challenges

#### Absence of consensus diagnostic criteria

One of the foremost challenges in CCS is the absence of universally accepted diagnostic criteria. Historically, the condition has been defined by the documentation of three periods of hypercortisolism separated by two periods of normocortisolism or hypocortisolism. Alternative definitions requiring only two peaks and one trough have also been used [1, 2]. The lack of standardization contributes to variability in reported prevalence and complicates comparisons across case series. It also increases the risk of underdiagnosis in patients whose cyclical pattern is incomplete at the time of evaluation.

#### Limitations of standard screening tests

Conventional first-line screening tests for Cushing’s syndrome — UFC, late-night salivary cortisol, and the 1-mg overnight dexamethasone suppression test — are less reliable in CCS because results depend heavily on the phase of the cycle at the time of testing [7]. Patients evaluated during a trough phase may present entirely normal results despite clear clinical features, whereas those assessed during a peak will fulfil biochemical criteria for Cushing’s syndrome. This temporal variability necessitates repeated testing to capture phases of hypercortisolism.

#### Role of dynamic testing and advanced strategies

Dynamic testing, such as the high-dose dexamethasone suppression test or the CRH stimulation test, may yield misleading results in CCS if performed during a trough phase, potentially leading to misclassification of the etiology [1, 7].

To improve diagnostic accuracy, experts recommend longitudinal biochemical surveillance with frequent sampling — such as serial late-night salivary cortisol over several weeks — or the use of continuous urinary steroid profiling by mass spectrometry, which can detect subclinical oscillations [2].

In ACTH-dependent forms, particularly those suspected to be ectopic, advanced localization strategies — including functional imaging (e.g., [^68Ga]-DOTATATE PET/CT) and bilateral inferior petrosal sinus sampling — are most informative when performed during hypercortisolemic phases. If initial studies are inconclusive, repeating them at a different phase of the cycle may increase sensitivity [3, 8].

#### Diagnostic delays and misclassification

The intermittent nature of hypercortisolism in CCS can lead to significant diagnostic delays, often spanning months to years, because early evaluations may be inconclusive [1, 2]. During trough periods, the absence of biochemical confirmation may be misinterpreted as exclusion of Cushing’s syndrome, resulting in misdiagnosis or attribution of symptoms to psychiatric, metabolic, or rheumatologic conditions [1]. Conversely, patients in peak phases may undergo unnecessary urgent interventions if the cyclical nature of their disease is not recognized.

These delays have direct clinical consequences: prolonged exposure to episodic hypercortisolism still result in cumulative morbidity, including osteoporosis, metabolic syndrome, cardiovascular disease, and increased susceptibility to infection [9, 10]. Recognizing the possibility of a cyclical pattern and maintaining a high index of suspicion, particularly in cases with fluctuating or relapsing symptoms, is therefore critical for timely diagnosis and intervention.

### Therapeutic strategies

#### When to treat? - The timing paradox

Therapeutic management of CCS is particularly complex due to fluctuating cortisol levels, unpredictable disease progression, and variable responses to interventions. A central dilemma is the so-called “timing paradox”: whether to initiate treatment during a peak of hypercortisolism or to defer until biochemical remission.

Acting during a phases of hypercortisolism surge allows prompt reversal of life-threatening complications, but it may hinder assessment of treatment efficacy, since spontaneous remission phases can occur independently of the intervention, making it difficult to distinguish true therapeutic benefit from the natural cycle. Conversely, deferring treatment until a trough may provide a clearer evaluation of efficacy, but risks delaying urgently needed interventions, with potentially severe consequences for patient safety. In CCS due to ectopic ACTH–secreting NETs, where peaks often precipitate life-threatening complications, many experts advocate proactive intervention based primarily on clinical severity rather than biochemical consistency [2, 6].

#### Medical therapy: variable efficacy in a moving target

Medical management in CCS typically relies on steroidogenesis inhibitors (metyrapone, ketoconazole, osilodrostat), glucocorticoid receptor antagonists (mifepristone), and, in ACTH-dependent forms, pituitary-directed agents such as pasireotide or cabergoline. However, the cyclic nature of cortisol secretion leads to erratic therapeutic responses and challenging dose titration [11].

Patients may experience transient biochemical control during peaks with rapid rebound in troughs, making sustained suppression challenging. Frequent monitoring and individualized dose adjustments are therefore essential, though rarely sufficient to achieve long-term stability.

#### Surgical considerations: adrenalectomy in cyclical disease

When first-line therapy fails to achieve sustained biochemical control — particularly in severe, recurrent hypercortisolism of ectopic origin — bilateral adrenalectomy often emerges as a viable and potentially life-saving option. Surgical series show rapid resolution of hypercortisolism and improvement in hypertension, glucose intolerance, and muscle weakness for many patients [12]. A systematic review of more than 700 adrenalectomy cases reported surgical morbidity rates around 18%, with acceptable long-term outcomes in high-volume centers [11]. Nevertheless, complications such as adrenal crises and Nelson’s syndrome, as well as lifelong dependence on glucocorticoid replacement therapy must be anticipated and carefully managed [12].

#### Role of tumor-directed therapies

In NET-associated CCS, controlling the primary tumor may reduce both tumor burden and cortisol fluctuations. Surgical resection, when feasible, remains first-line and can normalize cortisol production. If resection is incomplete or disease is metastatic, somatostatin analogues (e.g., octreotide) and systemic chemotherapy regimens (e.g., CAPTEM, platinum-based therapies) may help reduce tumor activity [1, 6].

Interestingly, several reports describe temporal associations between the initiation of tumor-directed therapies and subsequent cortisol peaks, emphasizing the need for vigilant monitoring during treatment transitions. Radiotherapy may also provide adjunctive control of hormonally active tumor tissue in selected patients [1, 6].

## Discussion

The present case provides a compelling illustration of the diagnostic and therapeutic challenges posed by ectopic CCS. Over several years, the patient experienced recurrent episodes of severe hypercortisolism interspersed with periods of biochemical remission, consistent with the classical definition of CCS. Such fluctuations had a significant impact on diagnostic timing, therapeutic decision-making, and clinical outcomes.

From a diagnostic perspective, this case demonstrates that the cyclical pattern of cortisol secretion in CCS may only become evident after prolonged follow-up. Although the initial diagnosis of ACTH-dependent Cushing’s syndrome was rapidly established based on classical clinical features and confirmatory biochemical evidence, recognition of its cyclical nature emerged later in the disease course. Intermittent normalization of cortisol levels highlighted the importance of continued biochemical surveillance and raised suspicion for CCS. This observation is consistent with previous reports indicating that cyclical patterns often evolve over time and are best identified through longitudinal monitoring, which tends to complicate long-term management rather than the initial diagnostic process.

Therapeutically, the case reflects the well-described “timing paradox” in CCS, wherein interventions were often initiated reactively, in response to clinical decompensation during hypercortisolemic peaks. Medical management with metyrapone was employed intermittently, requiring frequent dose adjustments. Ketoconazole was contraindicated, and access to newer agents such as osilodrostat was limited. The fluctuating hormonal activity complicated the evaluation of treatment response to NET-directed therapies, including chemotherapy, somatostatin analogues, and radiotherapy.

One of the most instructive aspects of this case is the temporal correlation between therapeutic changes and cortisol cycling. Periods of tumor progression or modification of antitumor therapy frequently coincided with hypercortisolism exacerbations, suggesting a bidirectional relationship between tumor dynamics and ACTH secretion. This observation supports emerging hypotheses that tumor burden, receptor modulation, and treatment-induced cellular stress may all influence secretory patterns in NET-related CCS.

The cumulative burden of cyclical hypercortisolism was reflected in the patient’s extensive comorbidities, including osteopenia, recurrent infections, nephrolithiasis, thromboembolic events, and metabolic syndrome, all contributing to physical frailty. Ultimately, the decision to proceed with bilateral adrenalectomy was driven by the failure of both medical and oncologic therapies to provide sustained biochemical control. The procedure achieved biochemical remission, but the patient remained steroid-dependent and continued to require palliative care for cancer-related symptoms.

This case underscores the complexity of CCS management in real-world clinical practice and reinforces the need for individualized and multidisciplinary approaches capable of adapting to the disease’s cyclic and unpredictable course. Furthermore, it highlights the potential value of recognizing temporal patterns in hormone secretion and integrating this knowledge into the timing and selection of interventions, an approach that may improve patient outcomes and reduce morbidity in similar cases.

## Conclusions

This case exemplifies the multifaceted challenges of managing ectopic CCS, where fluctuations in cortisol secretion influence every stage of care, from diagnosis to therapeutic timing. The intermittent nature of the disease can obscure biochemical confirmation, delay localization of the ACTH source, and complicate interpretation of treatment effects.

In NET-related CCS, tumor progression and therapeutic interventions may themselves modulate hormonal output, underscoring the importance of ongoing biochemical and imaging surveillance [3].

Bilateral adrenalectomy remains a valuable option when medical and tumor-directed therapies fail to achieve sustained control, despite its inherent risks and the need for lifelong glucocorticoid replacement therapy [9, 12].

Multidisciplinary management — involving endocrinology, oncology, surgery, and supportive care — is essential to optimize outcomes. Recognition of cyclical patterns, proactive intervention during hypercortisolemic crisis, and individualized alignment of endocrine and oncologic strategies may improve prognosis and quality of life in patients with CCS [1, 9].
